# Neural entrainment is associated with subjective groove and complexity for performed but not mechanical musical rhythms

**DOI:** 10.1007/s00221-019-05557-4

**Published:** 2019-05-31

**Authors:** Daniel J. Cameron, Ioanna Zioga, Job P. Lindsen, Marcus T. Pearce, Geraint A. Wiggins, Keith Potter, Joydeep Bhattacharya

**Affiliations:** 1 Department of Psychology, Neuroscience and Behaviour, McMaster University, Hamilton, ON Canada; 2 School of Biological and Chemical Sciences, Queen Mary University of London, London, UK; 3 Department of Psychology, Goldsmiths, University of London, London, UK; 4 Center for Music in the Brain, Department of Clinical Medicine, Aarhus University, Aarhus, Denmark; 5 AI Lab, Vrije Universiteit Brussel, Brussels, Belgium; 6 School of Electronic Engineering and Computer Science, Queen Mary University of London, London, UK; 7 Department of Music, Goldsmiths, University of London, London, UK

**Keywords:** Musical rhythm, Neural entrainment, Groove, Complexity, Timing

## Abstract

Both movement and neural activity in humans can be entrained by the regularities of an external stimulus, such as the beat of musical rhythms. Neural entrainment to auditory rhythms supports temporal perception, and is enhanced by selective attention and by hierarchical temporal structure imposed on rhythms. However, it is not known how neural entrainment to rhythms is related to the subjective experience of groove (the desire to move along with music or rhythm), the perception of a regular beat, the perception of complexity, and the experience of pleasure. In two experiments, we used musical rhythms (from Steve Reich’s *Clapping Music*) to investigate whether rhythms that are performed by humans (with naturally variable timing) and rhythms that are mechanical (with precise timing), elicit differences in (1) neural entrainment, as measured by inter-trial phase coherence, and (2) subjective ratings of the complexity, preference, groove, and beat strength of rhythms. We also combined results from the two experiments to investigate relationships between neural entrainment and subjective perception of musical rhythms. We found that mechanical rhythms elicited a greater degree of neural entrainment than performed rhythms, likely due to the greater temporal precision in the stimulus, and the two types only elicited different ratings for some individual rhythms. Neural entrainment to performed rhythms, but not to mechanical ones, correlated with subjective desire to move and subjective complexity. These data, therefore, suggest multiple interacting influences on neural entrainment to rhythms, from low-level stimulus properties to high-level cognition and perception.

## Introduction

Western musical rhythms typically have hierarchical metrical structures that elicit the perception of a periodic ‘beat’ (Sethares 2007), to which listeners tend to synchronize movements. Groove, or the desire to move along with musical rhythms, is highly associated with the experience of pleasure (Madison et al. 2011; Janata et al. 2012; Witek et al. 2014), and has an inverted-U relationship (or Wundt curve; see Wundt 1874; Berlyne 1971; Margulis and Beatty 2008) with rhythmic complexity (i.e., syncopation), so that both high and low levels of syncopation produce less grove than a moderate level (Sioros et al. 2014; Witek et al. 2014). However, the cognitive and neural underpinnings of this subjective experience associated with musical rhythm are largely uncharacterized (Levitin et al. 2018; Senn et al. 2016).

The perception of rhythm and beat depend on both stimulus characteristics and endogenous mechanisms, as the perceived beat is not an objective property of rhythms, but also mentally constructed by the listener (London 2012). One purported neural mechanism of rhythm and beat perception is the entrainment of ongoing neural oscillations to regularities in stimulus rhythms (Large and Snyder 2009). Neural entrainment is driven in part by external stimuli: inter-trial phase coherence (ITPC), a measure of phase consistency of stimulus-locked brain responses across repetitions (Makeig et al. 2001), increases during and immediately after listening to isochronous sounds (Will and Berg 2007), and the spectral EEG power at the frequency of isochronous sound presentation is also increased during listening (Nozaradan et al. 2011). However, neural entrainment is also governed by endogenous processes: imagining an emphasis on every second or third event of an isochronous rhythm increases the spectral EEG power at the frequency of the internally generated (imagined) emphasis (Iversen et al. 2009; Nozaradan et al. 2011). Unsurprisingly, neural entrainment can enhance perception and behaviour. For example, entrainment to predicted onsets of rhythmic auditory stimuli (including speech) improves the perception of those stimuli by aligning the excitable phase of an oscillation with the timing of expected stimuli (Calderone et al. 2014; Henry and Obleser 2012, 2014; Lakatos et al. 2008; Peelle and Davis 2012; Riecke et al. 2018). Attention to rhythmic stimuli enhances neural entrainment to, and perception of, those stimuli (Lakatos et al. 2008; Calderone et al. 2014), and entrainment to rhythms is correlated with the predictability of rhythmic events and also with reaction times to those events (Stefanics et al. 2010). These perceptual, behavioural, and cognitive interactions with neural entrainment may be relevant to the subjective experience of musical rhythms (e.g., the pleasure, groove, beat strength, and perceived complexity associated with a rhythm), although that link is not currently well understood.

Perceived groove and pleasure in rhythms are highly correlated (Witek et al. 2014), and are associated with complexity [i.e., syncopation or the intentional shifting of temporal emphases away from expected, regular positions, in musical rhythms (Temperley 1999)]. Neural entrainment is influenced by complexity of rhythmic structure (Nozaradan et al. 2012), although the exact nature of the relationships between neural entrainment and either structural or perceived complexity are not yet understood. Since we expected both neural entrainment and the perception of groove to be influenced by rhythmic complexity and by top–down factors like endogenous attention, we hypothesized that neural entrainment may be positively associated with subjective perception of groove, pleasure (which tends to be correlated with groove), and complexity.

Interactions with cognition and perception notwithstanding neural entrainment is stimulus-dependent—in the absence of other factors (e.g., predictability, learning), stimuli with highly regular temporal structure entrain neural oscillations and those that are temporally irregular do not (e.g., Fujioka et al. 2012). Music (like speech) is a curious case in this context, as it often relies on regularity in its temporal structure (rhythm, beat, and meter), but its real-world performance is not usually perfectly precise. Performers introduce temporal variability to musical rhythms, and rather than being erroneous, this variability of human performance is an inherent part of music, preferred over mechanical rhythms (i.e., computer generated, with sub-millisecond precision: Hellmer and Madison 2015; Hennig et al. 2011; Räsänen et al. 2015). It is often believed that such expressive deviations from precise timing make the music more engaging and increase the listener’s desire to move with the music (Iyer 2002; Fitch 2016). For example, a behavioural study found that when listening to expressively timed music compared to mechanical, precisely timed music, participants tapped the beat at higher levels of the metrical hierarchy which corresponded more closely to the temporal structure suggested by music theory (Drake et al. 2000). To the degree that neural entrainment may increase with preference and meter perception (due to, for example, engagement, attention, and perceptual salience), neural entrainment to music may be greater when rhythms are human performed, containing the temporal variability of expressive timing that listeners enjoy and use to infer meter. On the other hand, neural entrainment is also driven by temporal regularity (i.e., the opposite of variability) in a stimulus stream, which suggests that rhythms that are more precisely regular (i.e., that lack the timing variability of human performance) will elicit greater neural entrainment. Thus, manipulating the temporal precision of rhythms may reveal positive relationships between neural entrainment to rhythms and expressive timing (driven by subjective factors such as preference or beat perception), or negative relationships (driven by reduced temporal precision in human performed stimuli).

Here, we measured neural entrainment, as measured by ITPC, during listening to a piece of rhythmic music, Steve Reich’s *Clapping Music* (1972), that contains 12 distinct rhythms of varying complexity, as measured by the normalized pairwise variability index, nPVI (Patel and Daniele 2003). Critically, listeners heard two versions of the music: a mechanical version created digitally with precise timing, and a performed version with expressive timing natural to human performance. We recorded EEG from musicians, while they listened to *Clapping Music* and analyzed the ITPC values in the delta band (1–4 Hz), which contains the frequencies associated with the perceived beat and metrical structure. From a separate group of musicians, we collected ratings for each rhythm on four subjective measures of musical rhythm: the desire to move in time with the rhythm, the perception of a steady beat, the perception of rhythmic complexity, and the experience of pleasure. We hypothesized that neural entrainment to musical rhythms is positively associated with these aspects of subjective perception. Thus, we predicted positive correlations between ITPC and behavioural ratings of groove, complexity, pleasure, and beat for the individual rhythms of *Clapping Music*. Although complexity (e.g., syncopation) has been shown to have an inverted-U relationship with groove and pleasure, the rhythms from *Clapping Music* were expected not to reach the highest levels of syncopation used in the previous studies (e.g., Witek et al. 2014), and therefore, we predicted a positive linear relationship between ITPC and complexity. We also predicted that all four subjective ratings would be higher for rhythms with expressive timing of human performance than for the mechanical versions. Finally, we expected that listeners’ relatively greater engagement and attention associated with performed, compared to mechanical, rhythms would lead to stronger correlations between neural entrainment and subjective ratings.

We measured neural entrainment only in the delta band (1–4 Hz), a frequency range that excludes the rate of the minimum inter-onset intervals in the stimulus (5.33 Hz), which is prominent in the rhythms but faster than listeners tend to perceive as the beat. We examined the delta band only, as it contains the frequencies of the metrical regularities that are most important for the perception of beat and meter, and to which movements tend to synchronize during music listening. Relatedly, our focus on beat and meter-related frequencies partly explains why we predicted a positive relationship between perceived complexity and neural entrainment (i.e., in contrast to the faster stimulus regularity, for which we would expect a negative relationship). The degree of rhythmic complexity in *Clapping Music* was not thought to be so high as to inhibit beat perception or groove for any particular rhythm, or to substantially reduce stimulus-driven neural entrainment to the beat- and meter-related frequencies that are still present in the most complex stimuli.

## Materials and methods

### Experiment 1

#### Participants and stimuli

Twenty (14 female, mean age 25 years, range 19–39 years) trained, active musicians (with minimum 5 years of formal music training) participated in the EEG experiment. All participants gave written informed consent and received financial compensation for their participation. The experimental protocols were approved by the local ethics committee at Goldsmiths, University of London. All participants reported being unfamiliar with the musical source of the experimental stimuli, *Clapping Music.*

The stimulus was Steve Reich’s *Clapping Music*, a piece of contemporary classical music containing 12 distinct rhythms created by the application of a simple process of repetition and transformation applied to a basic rhythm by two performers clapping. Each rhythm has 12 metrical positions at which a single clap, two simultaneous claps, or a rest (silence) can occur. In common practice, each of the 12 rhythms is played 12 times sequentially before the transition to the next rhythm. The piece closes with the initial rhythm, played 12 times.

**Fig. 1:**
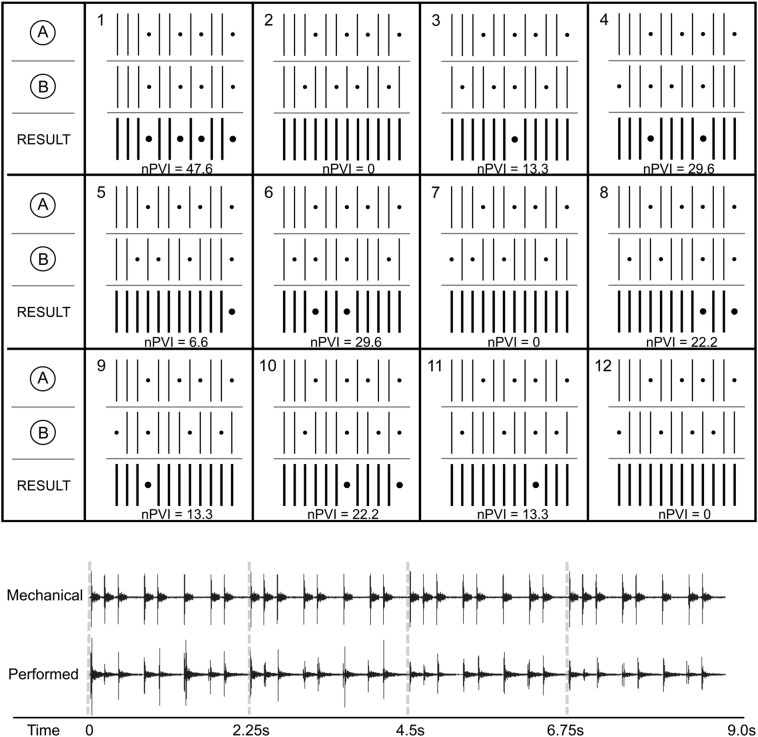
Top: The 12 unique rhythms from *Clapping Music* used as stimuli. Vertical lines indicate time positions at which a clap occurs. Dots indicate rests. For each rhythm, the two component rhythms (performed by different people clapping, labeled A and B) are shown, as well as the resultant rhythm, in darker lines and dots. Normalized Pairwise Variability Index (nPVI) values are included for each rhythm. This part of the figure is adapted from a figure in Cameron et al. (2017). Bottom: Waveforms of four repetitions of mechanical and performed versions of rhythm 1. Dashed grey lines indicate the onset of individual repetitions

We presented two versions of *Clapping Music*. First, a version with expressive timing (the *performed version*): a commercial recording of the piece (Reich 1987) performed by two individuals clapping, digitally manipulated to have a slower tempo and to have each repetition begin precisely 2.25 s after the onset of the previous repetition. Second, a version with mechanical timing (the *mechanical version*): created in MIDI using five samples from the commercial recording for each of five types of clapping sounds that account for the structure of the composition and performance instructions of the composer, specifically that performers should emphasize claps that occur on the downbeat in each rhythm. The five clapping sounds are: Performer 1 downbeat, Performer 1 non-downbeat, Performer 2 non-downbeat, both performers downbeat, and both performers non-downbeat. Clapping samples were placed in time exactly according to the notated intention of the composer (eliminating the subtle expressive shifts in intensity and timing that occur with human performance). The two versions, in full, are each 351 s in duration, presenting the 12 iterations of each of the 12 rhythms in continuous sequence (there is no gap between rhythms). See Fig. 1 for a representation of the 12 unique rhythms and waveforms of mechanical and performed versions of one rhythm. The two versions differed in stimulus intensity. Mechanical rhythms were slightly louder than performed rhythms (by 6.67 average momentary loudness units relative to full scale).

To obtain an objective measure of rhythmic complexity for each individual rhythm (as notated in the original score), we calculated normalized Pairwise Variability Index, nPVI (Grabe and Low 2002; Patel and Daniele 2003), a measure of the relative durational variability of a sequence (e.g., a rhythm):

$${\text{nPVI}} = \frac{100}{m - 1} \mathop \sum \limits_{k = 1}^{m - 1} \left| {\frac{{d_{k} - d_{k + 1} }}{{(d_{k} + d_{k + 1} )/2}}} \right|,$$

where *m* is the number of events in the rhythm and *d*_*k*_ is duration of the *k*th event.

#### EEG procedure, data collection, preprocessing, and analysis

Participants were presented with the full excerpt of the mechanical and performed versions once each (order counterbalanced across participants); participants were instructed to listen attentively to the music with eyes closed. Stimuli were played through two external speakers placed approximately 40 cm directly in front of participants.

We recorded EEG from 28 Ag/AgCl scalp electrodes placed according to the 10–20 system using electrode AFz as ground. Eye movements were recorded from electrode pairs placed around the eyes. All electrode impedances were kept below 5 kΩ. EEG signals were amplified (Synamps Amplifiers, Neuroscan Inc.), filtered (dc to 100 Hz), and sampled at 500 Hz. The average of two earlobe electrodes was used as reference. Data were epoched in 156 segments of 2.25 s length (1125 samples) and aligned with each of the 12 repetitions of the 13 rhythmic figures. Artifacts were detected by visual inspection and excluded from further analysis (< 0.5%). Ocular artifacts were removed by Independent Component Analysis using the EEGLAB toolbox (Delorme and Makeig 2004).

For each 2.25 s segment, we zero-padded an additional 5.75 s and applied a 4000-point fast Fourier transform (FFT) to obtain the amplitude (complex modulus of the FFT) and the phase (angle of the FFT) at individual frequencies, resulting in a frequency resolution of 0.125 Hz. The phase consistency over repetitions of the rhythm was measured by inter-trial phase coherence, ITPC (Makeig et al. 2001; Tallon-Baudry et al. 1996), varying between 0 (no consistency) and 1 (perfectly phase consistent across repetitions of the rhythm). ITPC was averaged across the frequencies of the delta band (1–4 Hz).

We used ITPC as an index of neural entrainment rather than spectral power, which has been used elsewhere (e.g., Will and Berg 2007; Henry and Obleser 2012; Doelling and Poeppel 2015). While spectral power may reflect neural entrainment, it is also sensitive to the amplitude of evoked potentials that occur at regular intervals. Because ITPC is more reliably related to neural entrainment than spectral power is (see Rajendran and Schnupp 2019; Haegens and Golumbic 2018; Zoefel et al. 2018), and also for the more specific reason that our stimuli differed in terms of intensity, which would lead to differences in amplitude of evoked responses.

Repeated-measures analysis of variance (ANOVA) was performed on delta-band ITPC values with the factors, Version (mechanical vs. performed) and Rhythm (1–13). Mean ITPC values for each rhythm, in each version, were then tested for correlation with the mean behavioural ratings for those same rhythms which were obtained in Experiment 2.

### Experiment 2

#### Participants and stimuli

Twenty-two (17 female, mean age 25 years, range 19–32 years) trained, active musicians (with minimum 5 years of formal music training) participated in this behavioural experiment. The two samples were independent as no participant took part in both experiments. All participants gave written informed consent and received financial compensation for their participation. The experimental protocols were approved by the local ethics committee at Goldsmiths, University of London. All participants reported being unfamiliar with *Clapping Music.*

Stimuli were taken from the same mechanical and performed version of *Clapping Music* as described above for Experiment 1. Rather than the full piece of music, participants were presented with four repetitions each of the individual rhythms (9 s), in each version (mechanical and performed).

#### Behavioural procedure, data collection, and analysis

For each rhythm, in each of the two types, participants provided ratings on a 7-point Likert scale, to four questions about their experience of the rhythm as follows:

1. Complexity: “How complex is this rhythm? 1 = extremely simple; 7 = extremely complex”
2. Pleasure: “How much pleasure do you experience listening to this rhythm? 1 = I experience no pleasure from this rhythm; 7 = I experience a great deal of pleasure”
3. Beat perception: “How strong was your sense of ‘beat’ in this rhythm? 1 = My sense of a regular ‘beat’ was extremely weak; 7 = My sense of a regular ‘beat’ was extremely strong”
4. Groove perception: “How much did this rhythm make you want to move? 1 = This rhythm did not make me want to move at all; 7 = This rhythm gave me a strong urge to move”.

Stimulus order was randomized individually for each participant, and rhythms were presented via headphones and ratings were collected via a laptop keyboard.

Ratings for each of the four questions for mechanical and performed rhythms were compared using one-tailed paired *t* tests, as we expected higher ratings for performed than mechanical rhythms.

#### Analyses comparing results from Experiments 1 and 2

To investigate the relationships between subjective evaluation of musical rhythms and neural entrainment, we calculated the Spearman rank correlation coefficient (*ρ*) between average ITPC values (i.e., averaged across participants) for each of the 12 rhythms, and the average rating for those same rhythms, for mechanical and performed versions separately. As we predicted positive correlations between ITPC and subjective ratings, we used one-tailed tests. To test whether listeners’ perception of rhythmic complexity is associated with an objective measure of rhythmic complexity, we tested the correlation between complexity ratings and nPVI. Finally, to test whether neural entrainment is associated with objective rhythmic complexity, we tested for correlation between ITPC and nPVI. Given the absence of data (to our knowledge) on the relationship between nPVI and perceived complexity in rhythms, and given the conflicting potential influences of rhythmic complexity on neural entrainment (i.e., stimulus- and perception-dependent factors as discussed above), correlation tests between nPVI and complexity ratings, and between nPVI and neural entrainment, were two-tailed.

We also calculated Spearman’s *ρ* between perceptual ratings for mechanical and performed rhythms separately, predicting positive correlations between all pairs of (1) complexity, (2) pleasure, (3) beat, and (4) groove (therefore, we used one-tailed tests).

All correlation tests were corrected for multiple comparisons using the false discovery rate (FDR: Benjamini and Hochberg 1995), separately for the correlations between ITPC, ratings, and nPVI, and the correlations between the different individual ratings (e.g., between groove ratings and complexity ratings, for mechanical and performed rhythms separately).

## Results

### Experiment 1

**Fig. 2:**
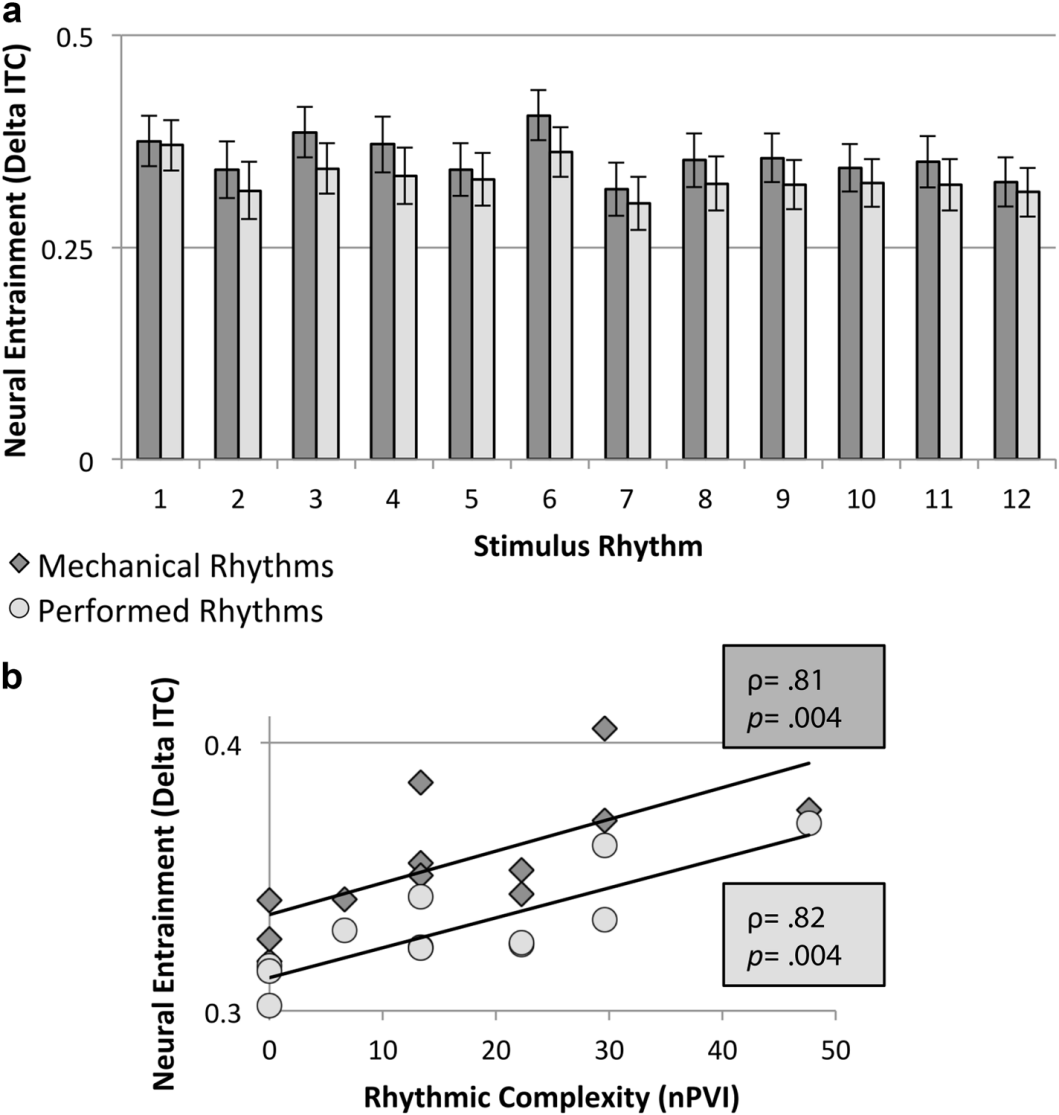
**a** Mean neural entrainment (ITPC) in the delta band of EEG across participants during listening to mechanical (dark bars) and performed (light bars) versions of the 12 rhythms of *Clapping Music.* Mean entrainment was greater for mechanical than performed rhythms. **b** Mean delta-band neural entrainment (ITPC) and normalized Pairwise Variability Index (nPVI) values (higher nPVI values indicate greater durational variability, taken as an objective measure of rhythmic complexity) for each rhythm. Dark squares are mechanical rhythms and light circles are performed rhythms. Entrainment and objective rhythmic complexity are positively correlated (i.e., ITPC correlates positively with nPVI). For the correlation, *p* values are two-tailed, FDR-corrected for multiple comparisons

Mechanical versions of rhythms elicited greater delta-band entrainment, as measured by ITPC, than performed versions (main effect of Version: *F*(1,19) = 23.62, *p* < 0.001, *η*^*2*^ = 0.55): mean entrainment to the mechanical version of each individual rhythm was higher (i.e., higher ITPC) than for the performed version (Fig. 2a). Individual rhythms differed in the degree of entrainment which they elicited (main effect of Rhythm: *F*(1,19) = 8.51, *p* < 0.001, *η*^*2*^ = 0.31). Rhythms with greater objective complexity (i.e., higher nPVI) elicited greater entrainment (i.e., higher ITPC) for both mechanical (Spearman’s rank correlation coefficient, *ρ* = 0.81, *p* = 0.001) and performed rhythms (*ρ* = 0.82, *p* = 0.001). The three rhythms with least objective complexity (or lowest nPVI; rhythms 2, 7, and 12) have the lowest mean ITPC values; the low complexity in these rhythms is related to the fact that they have sound onsets in every possible metrical position (no rests). The relationship between nPVI and entrainment is shown in Fig. 2b.

### Experiment 2

Mean behavioural ratings did not differ between mechanical and performed versions of rhythms for any of the attributes (complexity, groove, pleasure, or beat; *p* > 0.05).

For both mechanical and performed rhythms, perceived complexity correlated with the subjective ratings of both beat strength (mechanical: *ρ* = 0.69, *p* = 0.024; performed: *ρ* = 0.72, *p* = 0.024) and groove (mechanical: *ρ* = 0.78, *p* = 0.024; performed: *ρ* = 0.61, *p* = 0.034). For performed but not mechanical rhythms, perceived complexity was also correlated with pleasure (*ρ* = 0.65, *p* = 0.026) and pleasure was correlated with beat strength (*ρ* = 0.68, *p* = 0.024). Between-rating correlations are shown in Table 1.

**Table 1:** Spearman correlations between mean subjective ratings across rhythms

Mechanical rhythms					Performed rhythms			
	Complexity	Pleasure	Beat	Groove	Complexity	Pleasure	Beat	Groove
Complexity	–				–			
Pleasure	0.363 n.s.	–			**0.649** *p* = **0.026**	–		
Beat	**0.691** *p* = **0.024**	0.157 n.s.	–		**0.716** *p* = **0.024**	**0.677** *p* = **0.024**	–	
Groove	**0.784** *p* = **0.024**	0.466 n.s.	0.228 n.s.	–	**0.611** *p* = **0.034**	0.431 n.s.	0.240 n.s.	–

Statistically significant (*p* < .05) correlations are indicated in bold

### Comparing neural and behavioural responses

**Fig. 3:**
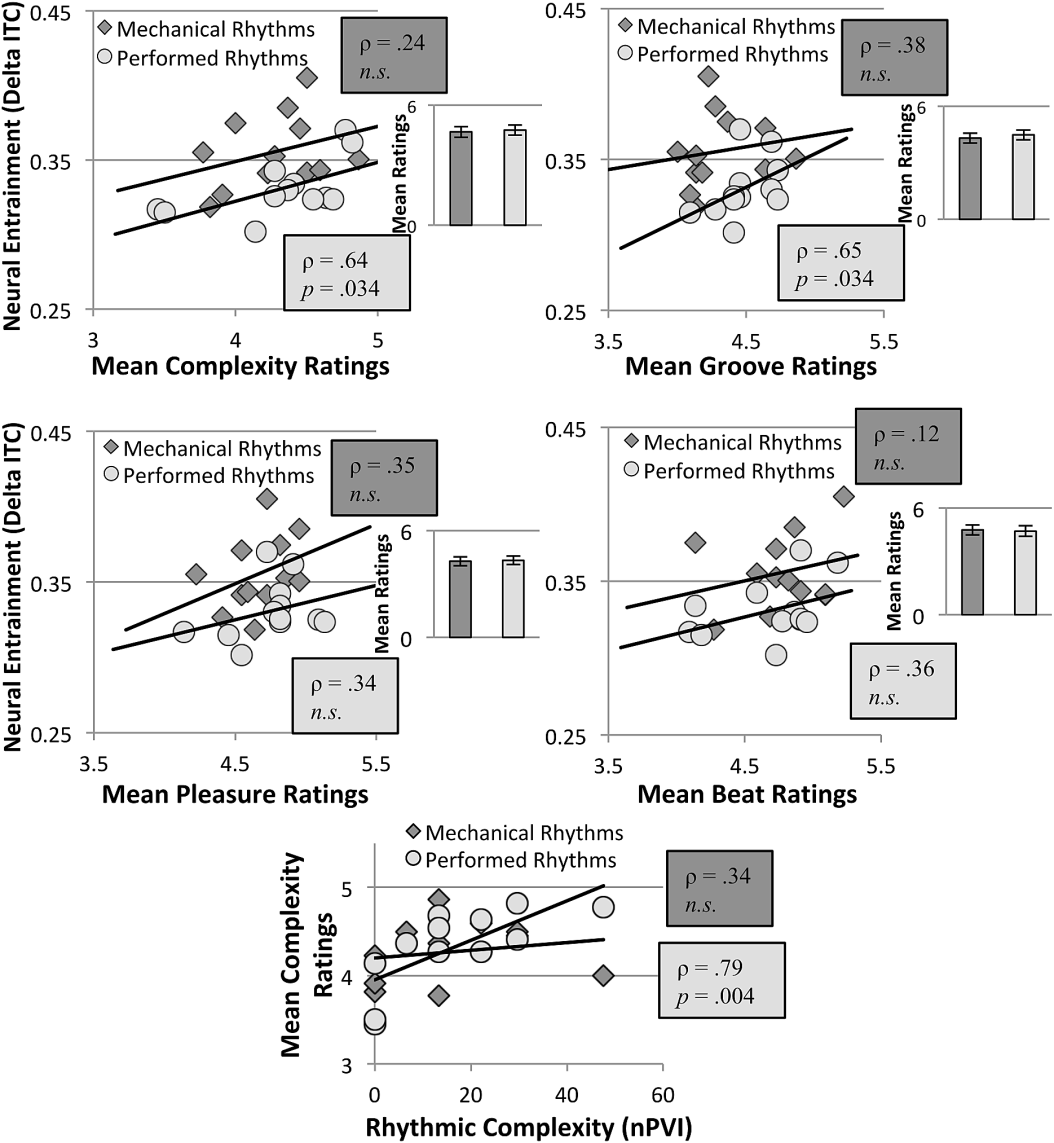
Neural entrainment (delta-band ITPC) and subjective ratings for perceptions of complexity (top left), induction to move (top right), pleasure (middle left), and beat strength (middle right), associated with mechanical (in darker shade) and performed (in lighter shade) rhythms. Spearman’s *ρ* and *p* values under 0.05 (one-tailed, FDR-corrected) are displayed for each condition in each chart. Neural entrainment to performed (but not mechanical) rhythms was found to correlate with perceived complexity and induction to move. Bar charts to the right of correlation figures show corresponding mean ratings across all rhythms; error bars indicate standard error of the mean. The bottom graph shows that objective rhythmic complexity correlates with perceived complexity of performed but not mechanical rhythms

Spearman correlations between neural entrainment (delta-band ITPC) and behavioural ratings were statistically significant only for performed rhythms for ratings of perceived complexity (*ρ* = 0.64, *p* = 0.034) and perceived groove (*ρ* = 0.65, *p* = 0.034) (see Fig. 3). In addition, ratings of perceived complexity correlated significantly with objective complexity (nPVI) for performed rhythms (*ρ* = 0.79, *p* = 0.004). Other correlations were not significant (*p* > 0.05).

## Discussion

Participants listened to mechanical (precisely timed) and human performed (with natural timing variability) versions of a piece of rhythmic music, *Clapping Music,* and their EEG was recorded to measure neural entrainment (the delta-band ITPC) to the 12 unique rhythms of the piece. A separate group of participants rated each of the rhythms, from mechanical and performed versions, and provided their subjective evaluations of rhythms on complexity, pleasure, beat, and groove. Neural entrainment, as measured by the delta-band ITPC, was greater when the rhythms were presented in a temporally precise, mechanical version compared to a performed version. We suggest that this difference in neural entrainment is likely due to the increased temporal precision of the mechanical rhythms—because the stimulus is more consistently timed, the entrained oscillations are more consistent, resulting in greater ITPC. However, overall subjective ratings of complexity, groove (the desire to move along to the rhythms), beat perception, and pleasure did not differ between performed and mechanical versions. Importantly, we observed relationships between neural entrainment and perceived complexity and groove only for the performed rhythms, but not for the precisely timed ones. Although subjective groove and complexity ratings did not differ between mechanical and performed rhythms, it may be that the functional relationships between neural entrainment and the experiences of subjective groove and complexity are different for the two types of rhythms. For example, it may be that the temporal variability of performed rhythms requires greater use of neural entrainment to attend to and assess the rhythms, or that when rhythms are perceptibly human-generated, subjective perception influences neural entrainment in a top–down fashion, possibly mediated by attention (i.e., a rhythm may be more salient when it is evidently produced by humans than if it is computer generated). It is further possible that the presence of relationships between neural entrainment and subjective perception for one but not the other stimulus type is due to different underlying influences on neural entrainment. This account is bolstered by the fact that both stimulus regularity (stronger for mechanical rhythms) and attention (possibly stronger for performed rhythms) can both increase neural entrainment (Fujioka et al. 2012; Lakatos et al. 2008; Calderone et al. 2014), but of those two factors, attention is more plausibly related to subjective perception. Alternatively, relationships between subjective perception of rhythms and neural entrainment to those rhythms could be present for mechanical rhythms but unobserved, because the relationship is so subtle that it is dominated by the relatively strong stimulus-driven component of neural entrainment. The present data cannot distinguish between the likelihood of these accounts (or others), so further research is needed to clarify the relationships between neural entrainment to stimulus characteristics and subjective perception of musical rhythms.

Groove, complexity (e.g., nPVI, syncopation), and pleasure have been shown to correlate with each other (Witek et al. 2014), and our behavioural ratings showed a similar pattern. Perceived complexity was correlated with groove for both mechanical and performed rhythms, while pleasure was correlated with complexity and beat only for performed rhythms. Pleasure and groove did not correlate for either version. The limited observed relationships between rated pleasure and groove, complexity, and neural entrainment may be due to the limited stimulus set (i.e., all short rhythms performed by clapping). The enjoyment of these particular rhythms was expected to be lower, and less variable, compared to the popular music recordings or drum kit performances used in the previous studies of groove (Janata et al. 2012; Witek et al. 2014). Perceived complexity (ratings) and objective complexity (nPVI) were only correlated for performed, and not for mechanical, rhythms, despite complexity ratings not differing between rhythm conditions. However, it is not clear why the relationship between objective rhythmic complexity (nPVI) and perceived complexity would be different for performed vs. mechanical rhythms.

Surprisingly, ratings did not differ between performed and mechanical rhythms. We expected that the expressive timing in performed rhythms would lead to higher ratings, particularly of pleasure and groove, based on the previous literature (Hellmer and Madison 2015; Hennig et al. 2011; Räsänen et al. 2015). It may be that the particular performed and mechanical stimuli used here are not sufficiently distinct in temporal variability to elicit differences in explicit ratings despite their different association with neural entrainment. This is consistent with other EEG research, showing that electrophysiological measures are, in some cases, more sensitive than behavioural measures (e.g., Francois and Schön 2011; Peretz et al. 2009). On the other hand, pleasure was significantly correlated with complexity and beat only in the expressive condition, suggesting that expressive timing may be a necessary condition for complexity and meter perception to exert influence on pleasure.

Neural entrainment was greater for rhythms with greater objective rhythmic complexity (nPVI). The three rhythms with lowest objective complexity (nPVI = 0; rhythms 2, 7, and 12) elicited the lowest levels of neural entrainment. Although this may seem contrary to the expected positive relationship between strict regularity in a stimulus and neural entrainment, it is worth noting again that we measured neural entrainment only in the delta band (1–4 Hz), a band that excludes the stimulus rate in these three isochronous rhythms (5.33 Hz).

It is worth considering our findings in the context of recent interest in entrained neural oscillations, its disentanglement from regularly occurring evoked neural responses, and its possible functions [see recent reviews by Zoefel et al. (2018), and Haegens and Golumbic (2018)]. We showed that phase locking in the delta-band EEG to rhythms depends both on objective properties of the rhythms and on subjective perception of them. We suggest that these results do not reflect differences in evoked responses arising after sound onsets in rhythms. Although ITPC was greater for the more precisely timed mechanical rhythms than for performed rhythms (a difference which could be due—at least in part—to sound-evoked responses that were more precisely regular), ITPC was not greater for the more structurally regular (less complex) rhythms, and, in fact, was greater for more complex rhythms when they were of the performed type. This suggests that differences in endogenous neural oscillations could contribute to the observed differences in ITPC.

Endogenous neural oscillations are thought to support temporal predictions (Lakatos et al. 2008; Arnal and Giraud 2012; Calderone et al. 2014; Zoefel et al. 2018), and can be driven by temporal predictability in stimulus streams. Here, we showed that neural entrainment was greater for more temporally regular (mechanical) rhythms, but also for more complex rhythms (both objectively and subjectively complex) if they were performed rather than mechanical. The correlations between subjective experience and neural entrainment to performed rhythms may support a recent proposal that complexity in musical rhythms is associated with bodily movement and pleasure by way of predictive neural mechanisms, and that beat-entrained movements not only elicit pleasure but aid sensory predictions (Vuust and Witek 2014). Thus, it may be that the predictive function of entrained endogenous oscillations supports neural and cognitive processing of complex rhythms, leading to the correlations between entrainment to performed rhythms and their perceived groove, and both objective and subjective complexity.

Subtle timing variation in rhythms, or micro-timing, may be related to the observed differences (and absence of differences) for performed and mechanical rhythms which differ in terms of temporal variability. The previous work has considered whether or not micro-timing in musical rhythms is related to groove, with mixed results (Butterfield 2010; Davies et al. 2013; Kilchenmann and Senn 2015). When micro-timing effects on groove have been shown, they tend to be related to systematic timing variation rather than ongoing variability arising unintentionally from natural human performance, and in relation to specific music genres. The lack of any difference in groove between the performed and mechanical conditions may be related to the fact that the stimuli are from a piece of music in a style not usually associated with groove (minimalist twentieth century art music), in the way that jazz and funk are, for example. Micro-timing may, however, be related to the differences between performed and mechanical rhythms in terms of relationships between neural entrainment and subjective perception of groove: micro-timing may impact attention, providing a functional link between neural entrainment and subjective perception, as discussed above.

Of note, the presentation order of rhythms was constant across EEG participants (the rhythms were presented as a musical composition to keep the ecological validity of musical listening), but differed across participants completing the behavioural experiment (randomized order of individual rhythms). While we do not expect that the order of rhythm presentation casts significant doubt on our main conclusions (as the order was the same for both mechanical and performed rhythms in the EEG experiment, and did not systematically differ between conditions in the behavioural experiment), a previous study showed that the rhythms (or rhythmic figures) of *Clapping Music* are more easily differentiated (rated as less similar) when heard within the context of the entire piece of music rather than as isolated pairs (Cameron et al. 2017). Therefore, it is possible that relationships between perceptual ratings and neural entrainment would be stronger if presentation order was the same in the two experiments (although this would either reduce ecological validity in the EEG experiment or risk introducing order effects on subjective ratings).

While we believe that the use of real music provides ecological validity to the study of rhythm perception and elicits greater engagement from participants, the need certainly exists to use a broader range of stimuli to investigate in further breadth and detail the relationships between neural entrainment to, and perception of, musical rhythms. For example, using a larger set of rhythms with a range of complexity (nPVI) that reached higher levels of complexity might replicate the correlation between complexity and neural entrainment observed here, but might, instead, reveal an inverted-U relationship between neural entrainment with complexity, as observed previously for groove and preference (Witek et al. 2014).

All participants were trained musicians in our two experiments; both behavioural and neural differences associated with rhythm and beat perception have been found between musicians and non-musicians (e.g., Drake et al. 2000; Grahn and Rowe 2009), including an enhancing effect of musical training on neural entrainment to music and rhythms (Doelling and Poeppel 2015; Stupacher et al. 2017). In addition, participants were primarily trained in Western music and were all living in the UK (i.e., as a sample they did not represent global cultural diversity), and learning, enculturation, and experience are known to influence both musical rhythm perception (Hannon and Trehub 2005; Hannon and Trainor 2007; Hannon et al. 2012; Stevens 2012; Cameron et al. 2015; Bouwer et al. 2018; Polak et al. 2018) and neural entrainment to music, rhythms, and speech (Doelling and Poeppel 2015; Stupacher et al. 2017; Song and Iverson 2018). Therefore, although the phenomena of interest (beat perception and tendency to entrain to musical rhythms) are found widely across the world, in all cultures, and do not require training, enculturation and training may influence, and thus limit the generalizability of, the observed relationships between the perception of and neural entrainment to musical rhythms.

Altogether, we demonstrate links between the subjective experience of, neural entrainment to, and complexity of performed (but not mechanical) musical rhythms. The causal links between these factors and measures remain to be understood—for example, neural entrainment could either cause or arise from the desire to move while listening to rhythms—but the presented results contribute to understanding the seeming magic that music exerts on our senses, bodies, brains, and lives.
